# Antimicrobial-resistant genes associated with *Salmonella* spp. isolated from human, poultry, and seafood sources

**DOI:** 10.1002/fsn3.119

**Published:** 2014-05-26

**Authors:** Yemisi O Adesiji, Vijaya Kumar Deekshit, Indrani Karunasagar

**Affiliations:** 1 Department of Fisheries Microbiology, Karnataka Veterinary Animal and Fisheries Sciences University, College of FisheriesMangalore, 575002, India

**Keywords:** Antimicrobial resistance, India, Nigeria, resistant genes, *Salmonella*

## Abstract

Antimicrobial-resistant salmonellosis is a significant public health concern globally. A study was conducted to screen for *Salmonella* species from a total of 120 samples, of which 50 were retail meat samples purchased from five randomly selected sales outlets in the city of Mangalore, India. Twenty poultry fecal materials freshly voided before slaughter were obtained with sterile spatula and placed in sterile sealable polythene envelopes, and 20 clams were purchased from the estuaries of Nethravathi and Kankarnady market. In addition, 30 clinical isolates from Nigeria suspected to be *Salmonella* by only cultural characterization were also included in the study. In all, 30 samples—6 poultry, 8 seafood, and 16 *Salmonella* isolates from clinical samples—were confirmed positive by PCR and used in this study. The disk-diffusion test was performed to determine the zone of inhibition, and detection of resistance genes was tested by PCR targeting various antimicrobial genes. Resistance to tetracycline (TET), cotrimoxazole, nalidixic acid, nitrofurantion, and piperacillin/tazobactin was found in 66.7%, 60%, 53.3%, 50% and 50% of the isolates, respectively. About 60–100% of MDR isolates possessed antibiotic-resistant genes, of the tetracyclines resistant isolates, 20 (100%) 6 (30%), 7 (35%), and 10 (50%) carried *tetA*, *tetB*, *tetC*, and *tetG* genes, respectively. Of 18 cotrimoxazole-resistant strains, 18 (100%), 14 (77.7%), and 4 (22.2%) had *sul1*, *sul2*, and *sul3* genes, respectively. Of the 14 multidrug-resistant isolates tested, 8 (61%) and 9 (69%) were positive for *cmlA* and *cmlB* genes, respectively, 10 (1.4%) tested positive for *aph*(3)11a genes, 8 (57%) tested positive for *aac(3)lla*, while none was positive for the *aac6* gene. The results show the presence of antibiotic-resistant *Salmonella* spp. in food samples from India and in human samples from Nigeria.

## Introduction

Salmonellosis encompasses a wide spectrum of diseases in humans and animals which may manifest as acute gastroenteritis, bacteremia, and extraintestinally localized infections involving many organs. Although intestinal infection caused by nontyphoid *Salmonella* serotypes is usually self-limiting, effective antimicrobial therapy is essential if spread beyond the intestine occurs (Dione et al. 2011).

The extensive use of antimicrobials in humans and animals has led to an increase in multidrug resistance among several bacterial strains. Multidrug-resistant (MDR) *Salmonella* strains have been among the major public health concerns worldwide, while sea food, chickens, and fish are known to be important reservoirs of *Salmonella* spp. (Bhowmick et al. 2009).

The incidence of resistance to antibiotics of bacteria originating from food animals or retail meat is on the rise in developing countries (Van et al. 2007), possibly as a result of the inappropriate or uncontrolled use of antibiotics in farming practices. Subsequent transmission of antimicrobial resistance to humans can be in the form of either resistant pathogens or commensal organisms carrying transferable resistance genes. However, a number of studies have investigated the assertion that the use of antimicrobial agents in animal production systems might lead to either a sustained increase in antimicrobial resistance among animal pathogens or the occurrence of drug-resistant pathogens in people (Heider et al. 2009; Mann et al. 2011; Morley et al. 2011).

Antibiotic resistance, especially to the most commonly used antimicrobials in humans and in animal production systems, is of critical concern in Nigeria where MDR *Salmonella* strains are among the most frequent causes of bacteremia in children (Fashae et al. 2010). *Salmonella* serotypes with reduced fluoroquinolone susceptibility from human have been documented (Akinyemi et al. 2007). A previous study which screened S*almonella* species from food handler and animal isolates showed that the fingerprinting observed shared the same RAPD patterns, which is indicative of the fact that the food handlers could have been infected from the animal sources as samples were collected from sites used by food handlers (Smith et al. 2011).

Thus, assessing the distribution of resistance genes in bacterial population represents a more detailed and potentially useful additional tool for improving our understanding of Antimicrobial resistance epidemiology, particularly in southwestern Nigeria where such information is limited. Therefore, this study intends to document the frequency and trends of antimicrobial resistance present in the *Salmonella* isolates from Nigeria and India.

## Methods

### Sample collection

A total of 120 samples were screened. Of these, 50 were retail meat samples purchased from five randomly selected sales outlets in the city of Mangalore. Twenty poultry fecal materials freshly voided before slaughter were obtained with sterile spatula and placed in sterile sealable polythene envelopes, and 20 clams purchased from the estuaries of Nethravathi and the Kankarnady market, Mangalore, were placed in a sterile bottle. In addition, 30 isolates of *suspected Salmonella* cultures (not previously confirmed by PCR) from collection in the Department of Medical Microbiology, University of Ilorin Teaching Hospital, Nigeria, were also screened.

### Bacterial isolation

*Salmonella* was isolated by the conventional method as per the protocols recommended by FDA and Andrew (1996). A sample weight of 25 g was homogenized with 225 mL of lactose broth for 2 min. The mixture was then incubated at 37°C for 24 h. One milliliter each of pre-enriched sample was added to 10 mL each of selenite cystine broth (SCB). The inoculated SCB was incubated at 37°C for 24 h. Subsequently a loop full of culture from each of these broths was streaked on Hektoen enteric agar (HEA) and xylose lysine deoxycholate agar (XLD) and incubated at 37°C for 24 h. Suspected colonies of *Salmonella* (minimum of five colonies) from each selective agar were subjected to a series of biochemical tests which include oxidase, catalase, IMViC (Indole, Methylred, Voges-Proskauer, and Citrate), TSIA (triple sugar iron agar test), LIA (lysine iron agar test), and urease test.

### Extraction of template DNA for PCR assay

One milliliter of bacteria grown overnight at 37°C in 5 mL of Luria–Bertani broth was dispensed aseptically in an Eppendorf tube. Bacterial genomic DNA was extracted using the cetyl trimethyl ammonium bromide (CTAB) method as described previously by Ausubel et al. (1992). The DNA quantity and quality was determined using a NanoDrop ND-1000 spectrophotometer (NanoDrop® Technologies, Thermo Scientific, Pittsburgh, PA) by measuring the absorbance at 260 nm and the ratio of *A*_260_/*A*_280_, respectively.

PCR was performed using genus-specific primers *hns* (Jones et al. 1993) and *invA* (Rahn et al. 1992). Briefly, the reaction was performed in 30 *μ*L volumes containing 3 *μ*L of 10× buffer (100 mmol/L Tris-HCl [pH 9], 1.5 mmol/L MgCl_2_, 50 mol/L KCl, and 1% gelatin), 50 *μ*mol/L of each of the four deoxyribonucleotide triphosphates (dATP, dGTP, dCTP, and dTTP), 10 pmol of each primer pair, and 1.0 U of *Taq* DNA polymerase with 2 *μ*L of template DNA. The optimized PCR conditions consisted of an initial denaturation at 95°C for 5 min followed by 35 cycles of denaturation at 95°C for 1 min, annealing at 55°C for 1 min, extension at 72°C for 1 min, and a final extension at 72°C for 10 min. The amplified products were resolved by electrophoresis on 2.5% agarose gel, stained with ethidium bromide, and visualized under UV light using a gel documentation system (Herolab, Wiesloch, Germany).

### Antimicrobial susceptibility testing

The antimicrobial susceptibility test was performed using the disk diffusion method as described by Bauer et al. (1966). Overnight-grown cultures in Luria–Bertani broth (HiMedia Laboratories Pvt. Ltd., Mumbai, India) were prepared in a lawn on Mueller Hinton agar. The antibiotics disks were placed aseptically on it and incubated at 37°C for 16–18 h. Clinical and Laboratory Standards Institute (CLSI) guidelines were used to interpret results (CLSI 2010). The following antimicrobials were used: ampicillin (AMP, 20 *μ*g), cefotaxime (CTX, 300 *μ*g), chloramphenicol (C, 30 *μ*g), tetracycline (TET, 30 *μ*g), ciprofloxacin (CIP, 5 *μ*g), gentamicin (GEN, 10 *μ*g), nalidixic acid (NA, 30 *μ*g), cotrimazole (COT, 25 *μ*g), tetracycline (TET), nitrofurantion (NIT, 30 *μ*g), imepenem (IPM, 10 *μ*g), meropanem (MRP, 10 *μ*g), piperacilin/tazobactin (PIT, 100/10). Antibiotics were manufactured by Himedia (Mumbai, India).

### Antimicrobial resistance gene detection

For detecting antimicrobial-resistant genes in 30 *Salmonella* isolates, target genes conferring resistance to tetracyclines (*tetA*, *tetB*, *tetC*, *tetD*, *tetE*, and *tetG*), sulfonamides (*sul1*, *sul2*, and *sul3*), chloramphenicol (*cat1, cat2, and cat3, cmlA, cmlB, floR*) and aminoglycosides (*aph(3)11a, aac(3)11a and aac6*) were screened by PCR with their respective primers. The cycling conditions and primer sequences were as described by Ma et al. (2007). The PCR was performed in 30 *μ*L volumes containing 3 *μ*L of 10× buffer (100 mmol/L Tris-HCl [pH 9], 1.5 mmol/L MgCl_2_, 500 mmol/L KCl, 0.1% gelatin), 100 *μ*mol/L concentrations each of dATP, dTTP, dGTP, and dCTP, 10 pmol of each primer, and 0.9 U of *Taq* DNA polymerase (Bangalore Genei, Bangalore, India), with 2.0 *μ*L of template DNA. The reactions were carried out using a thermal cycler (MJ Research, Bio-Rad, Hercules, CA). Primer sequence and cycling conditions are summarized in Table 1.

**Table 1:** Primer sequences and their annealing temperatures used in this study

Resistance gene	Primer	Nucleotide sequence 5′–3′	Product size (bp)	Annealing temperature (°C)	Code of antibiotics	References
*tetA*	F	TTGGCATTCTGCATTCACTC	494	55	TET	Ma et al. (2007)
	R	GTATAGCTTGCCGGAAGTCG				
*tetB*	F	CAGTGCTGTTGTGTCATTAA	571	55	TET	Ma et al. (2007)
	R	GCTTGGAATACTGAGTGTAA				
*tetC*	F	CTTGAGAGCCTTCAACCCAG	418	55	TET	Ma et al. (2007)
	R	ATGGTCGTCATCTACCTGCC				
*tetD*	F	GCTCGGTGGTATCTCTGCTC	546	55	TET	Ma et al. (2007)
	R	AGCAACAGAATCGGGAACAC				
*tetE*	F	TATTAACGGGCTGGCATTTC	544	55	TET	Ma et al. (2007)
	R	AGCTGTCAGGTGGGTCAAAC				
*tetG*	F	GCTCGGTGGTATCTCTGCTC	550	55	TET	Ma et al. (2007)
	R	CAAAGCCCCTTGCTTGTTAC				
*Sul1*	F	TTTCCTGACCCTGCGCTCTAT	793	55	COT	Ma et al. (2007)
	R	GTGCGGACGTAGTCAGCGCCA				
*Sul2*	F	CCTGTTTCGTCCGACACAGA	667	55	COT	Ma et al. (2007)
	R	GAAGCGCAGCCGCAATTCAT				
*Sul3*	F	ATGAGCAAGATTTTTGGAATCGTAA	792	55	COT	Ma et al. (2007)
	R	CTAACCTAGGGCTTTGGTATTT				
*cat1*	F	AACCAGACCGTTCAGCTGGAT	549	55	CHL	Zhao et al. (2001)
	R	CCTGCCACTCATCGCAGTAC				
*cat2*	F	AACGGCATGAACCTGAA	547	55	CHL	Ma et al. (2007)
	R	ATCCCAATGGCATCGTAAAG				
*cat3*	F	ATCGGCATCGGTTACCATGT	310	55	CHL	Ma et al. (2007)
	R	ATCCCCTTCTTGCTGATATT				
*cmlA*	F	GGCCTCGCTCTTACGTCATC	662	55	CHL	Ma et al. (2007)
	R	GCGACACCAATACCCACTAGC				
*cmlB*	F	ACTCGGCATGGACATGTACT	840	55	CHL	Ma et al. (2007)
	R	ACGGACTGCGGAATCCATAG				
*floR*	F	ATGACCACCACACGCCCCG	198	55	CHL	Ma et al. (2007)
	R	AGACGACTGGCGACTTCTTCG				
*aac(3)11a,*	F	CGGCCTGCTGAATCAGTTTC	439	55	GEN	Ma et al. (2007)
	R	AAAGCCCACGACACCTTCTC				
*aph(3)11a*	F	TCTGAAACATGGCAAAGGTAG	582	55	GEN	Ma et al. (2007)
	R	AGCCGTTTCTGTAATGAAGGA				
*aac6*	F	TTGGACGCTGAGATATATGA	476	55	GEN	Ma et al. (2007)
	R	GCTCCTTTTCCAGAATACTT				
blaTEM-1	F	CAGCGGTAAGATCCTTGAGA	643	55	Control	Ma et al. (2007)
	R	ACTCCCCGTCGTGTAGATAA				
16S rDNA	F	AGAGTTTGATCMTGGCTCAG	907	55	Control	Ma et al. (2007)
	R	CCGTCAATTCMTTTRAGTTT				

TET, tetracycline; GEN, gentamicin; COT, cotrimazole.

## Results

**Figure 1:**
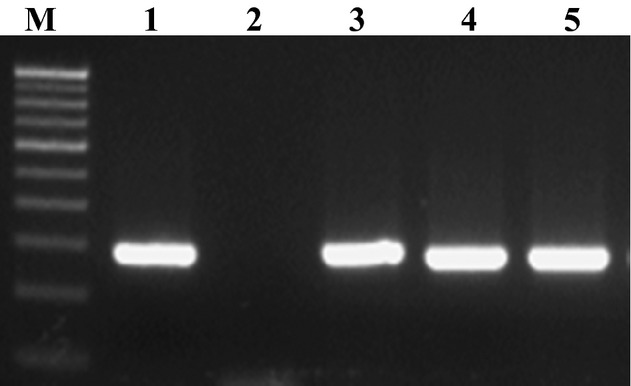
PCR amplification of *invA* gene. Lane M: 100 bp DNA Ladder (Genei TM, Merck Bangalore) Lane 1: Positive control (ATCC 14028) Lane 2: Negative control Lanes 3-5: Samples positive for *Salmonella* spp.

**Figure 2:**
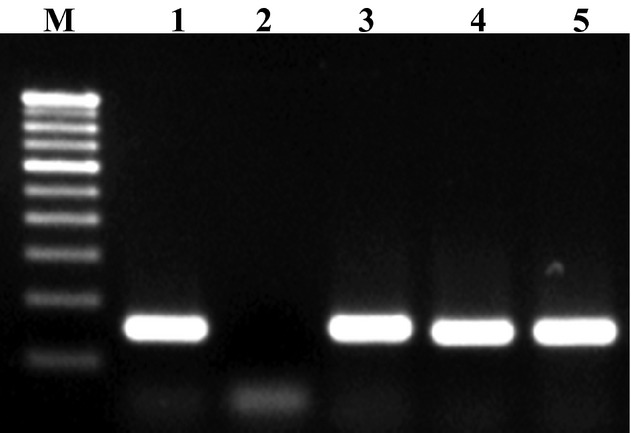
PCR amplification of *hns* gene. Lane M: 100 bp DNA Ladder (Genei TM, Bangalore) Lane 1: Positive control (ATCC 14028)Lane 2: Negative control Lanes 3-5: Samples positive for *Salmonella* spp.

**Figure 3:**
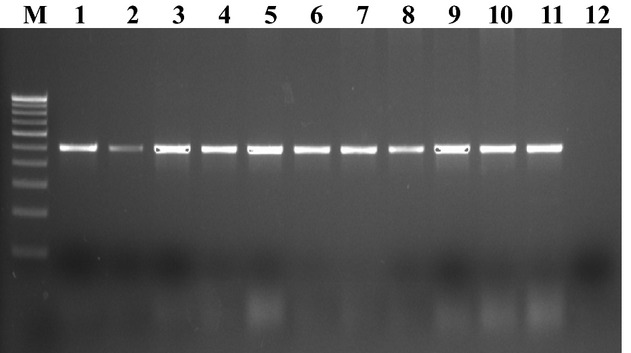
Showing representative *Tet A* gene from S*almonella*. Lane M- 100bp marker, Lane 2: Positive control, Lanes 3-11: test samples and Lane 12: negative control.

**Figure 4:**
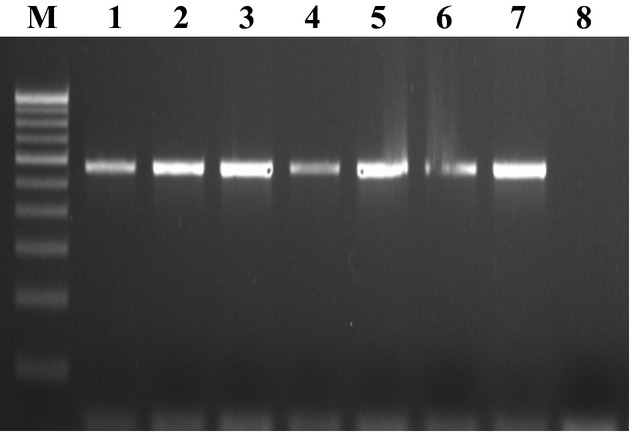
Showing representative *TetB* gene from S*almonella*. Lane M- 100bp marker, Lane 2: Positive control, Lanes 3-11: test samples, Lane 8: negative control.

All the isolates used in the study were confirmed as *Salmonella* by PCR amplification of the *hns* and *invA* genes, which generated amplicons of 152 and 284 bp, respectively (Figs. 1 and 2). A total of 30 samples were confirmed positive for *Salmonella* by conventional as well as by molecular methods. Six *Salmonella* isolates from poultry, eight from seafood, and 16 from clinical samples were used in this study. Resistance to TET, cotrimoxazole, NA, NIT, and piperacillin/tazobactin was found in 20 (66.7%), 18 (60%), 16 (53.3%), 15 (50%) and 15 (50%) of the isolates, respectively. Resistance to chloramphenicol, CTX, AMP, and GEN was also detected in 20–10% of the isolates. About half (50.5%) of the isolates were resistant to at least one antibiotic. All of the 20 TET-resistant isolates carried *tetA* gene and 30% (6), 35% (7), and 50% (10) of the isolates carried *tetB*, *tetC*, and *tetG* genes, respectively. Figs 3 and 4 shows the plate of representative sample of *tetA* and *tetB* genes recovered from the isolates. Of 18 cotrimoxazole-resistant strains, 18(100%), 14 (77.8%), 4 (22.2%) had *sul1*, *sul2*, and *sul3* genes, respectively. Table 2 shows the summary of resistant pattern and genes from all isolates. Six isolates were resistant to chloramphenicol, but more isolates (10 of 14 multidrug resistant) were positive for *floR* and *cat 2* genes, while 2 (30%) was positive for *cat* 3 genes. Of 14 multidrug-resistant isolates tested 8 (61%) and 9 (69%) were positive for *cmlA* and *cmlB* genes, respectively.

**Table 2:** Antimicrobial resistance and resistant gene profiles of *Salmonella* isolates from retail raw food obtained from India and clinical isolates from Nigeria

Isolates	Antimicrobial resistance profile	Antimicrobial-resistant gene(s)	Sample source	Country
Sal 6	TET-PIT-NIT	*tet A*, *tetD*, *tetG*, *sul* 1, *cat2*, *cat 3*, *cmlB*	Clinical	Nigeria
Sal 8	TET-COT-C-CTX-AMP	*Tet A*, *tetB*, *tetC*, *tet G*, *Sul1*, *sul2*, *Cat1*, *Cat2*, *Cat 3*, *floR*, *aac(3)11a*, *aph(3)11a*	Clinical	Nigeria
Sal 10	TET-COT-C-AMP	*tet A*, *tet B*, *tet C*, *tet C*, *sul1*, *sul2*, *cat1*, *cat2*, *floR*, *aac(3)11a*, *aph(3)11a*	Clinical	Nigeria
Sal 14	TET-COT-C-CTX-AMP	*Tet A*, *tet B*, *tet C*, *sul1*, *cat1*, *cat 2*, *cmlA*, *cmlB*, *floR*, *aac(3)11a*, *aph(3)11a*	Clinical	Nigeria
Sal 15	TET-COT-GEN-NA-C-CTX-AMP	*tet A*, *tet B*, *sul1 cat1*, *cat*2, *cmlA*, *cmlB*, *floR*, *aac(3)11a*, *aph(3)11a*	Clinical	Nigeria
Sal 16	TET-COT-GEN-CTX-AMP	*tet A*_,_ *tet B*_,_ *tet C*_,_ *Sul 1*, *Sul2*, *Cat2*, cmlA, cmlB, floR, *aac(3)11a*, *aph(3)11a*	Clinical	Nigeria
Sal 17	TET-NA-NIT	*tet A*, *TetD*, *floR*	Poultry	India
Sal 18	MRP-TET-COT-NA-NIT	*tet A*, *Tet C*, *sul*1, *Cat2 cmlA*, *floR*, *aac(3)11a*, *aph(3)11a*	Poultry	India
Sal 19	TET-COT-NIT	*tet A*, *Tet C*, *tetD*, *Sul1*, *floR Cat2*	Poultry	India
Sal 20	TET-COT-NA-NIT	*tet A*, *tetD*, *tetG*, *sul* 1, *Sul2*, *cat2*, *cmlB*	Poultry	India
Sal 21	MRP-TET-CIP-PIT-GEN-NA, CTX-AMP-NIT	*tet A*, *tet C*, *Tet D*, *Sul 1*, *cat1*, *cat3*, *aac(3)11a*, *aph(3)11a*	Poultry	India
Sal 22	MRP-TET-NA-CTX-AMP-NIT	*tet A*, *sul1*, *cat1*, *cat3*, *aac(3)11a*, *aph(3)11a*	Poultry	India
Sal 25	TET-COT, PIT, NA, NIT	*tet A*, *sul1*, *sul2*, *sul3*, *cat1*, *cmlA*, *cmlB*, *floR*	Clam	India
Sal 30	TET, COT, PIT, NA, NIT	*tet A*, *Sul 1*, *sul2*, *cat1*, *cmlA*, *cmlB*, floR	Clam	India

Amp, ampicillin; CTX, cefotaxime; TET, tetracycline; CIP, ciprofloxacin; GEN, gentamicin; NA, nalidixic acid; COT, cotrimazole; NIT, nitrofurantion; IPM, imepenem; MRP, meropanem; PIT, piperacilin/tazobactin.

## Discussion

Monitoring antimicrobial resistance trends among bacteria isolated from food, animals, and humans is necessary to inform public policy regarding the appropriate use of antimicrobial agents in veterinary and human medicine (Cummings et al. 2013). Some studies conducted in Nigeria also indicated considerable prevalence of *Salmonella* both in veterinary and clinical samples (Fasure et al. 2012; Ogunleye et al. 2013).

In this study, high levels of resistance were found to trimethoprim-sulfamethoxazole, TET, and GEN; 66.7%, 60%, 53.3%, and 50%, respectively. A comparative study in Ibadan, Nigeria, reported a high frequency (87%) of reduced susceptibility to CIP among the chicken isolates and a high frequency of resistance to TET (93%), NA (81%), and sulfamethoxazole (87%), while resistance to chloramphenicol, sulfamethoxazole, trimethoprim, and AMP ranged from 36% to 59% for the human isolates (Fashae et al. 2010). In another study, 100% resistance to fluoroquinolones from clinical isolates from northern part of Nigeria was reported (Akyala et al. 2013). Only one poultry isolate from chicken was resistant to CIP in the present study, but reduced susceptibility was observed for clinical isolates; and all clam isolates from India were resistant to NA while in contrast Fashae et al. (2010) in his previous study reported that four *Salmonella* Derby isolates from their chickens showed reduced susceptibility to CIP and high susceptibility to NA. High level of resistance to NA 16 (53%) as observed in this study particularly from food animal reaffirms the importance of the need for strengthening collaboration between veterinary and public health sectors on appropriate detection and reporting of zoonotic foodborne pathogens (Adesiji and Fagbami 2006). In addition, the result obtained from this study is of high significance because treatment with antimicrobials is crucial for the proper management of severe or invasive human salmonellosis. Fluoroquinolones and third-generation cephalosporins are now commonly used in adults for treatment due to widespread resistance to chloramphenicol, AMP, and cotrimoxazole. Fluoroquinolones are often the last resort for treatment of children and are listed by the World Health Organization as critically important antimicrobials for human health (Collignon et al. 2009). Prescription pattern, availability, and cost-effectiveness of quinolones as drugs that are usually prescribed in the management of most resistant bacterial infections were suggested as factors that could be responsible for continued rapid evolution of fluoroquinolone-resistant bacteria in Nigeria (Lamikanra et al. 2011). The limitation of this study was its inability to compare human and clinical isolates from the same country setting due to the fact that all veterinary samples suspected to be *Salmonella* were not confirmed positive by PCR. Resistance to traditional antibiotics (AMP, TET, and SUL) was high in *Salmonella* isolates from animals and foods of animals as observed in this study, previously reported by Deekshit et al. (2012). It is apparent that resistance to traditional antibiotics such as TETs, AMPs, and cotrimoxazole and detection of their genes in microbial populations of both countries constitute a public health concern by limiting the therapeutic choices for treating salmonellosis in animals and humans. More so, in most developing countries such as Nigeria and India, many of these antibiotics are also used commonly in human therapy due to their low cost and ready availability (Wannaprasat et al. 2011). TET-resistant genes occur most frequently in our study, all of the 20 TET-resistant isolates carried *tetA* gene and 30% (6), 35% (7), and 50% (10) of the isolates carried *tet B*, *tet C*, and *tet G*, genes, respectively. TET-resistant genes were also detected in most TET-susceptible and resistant isolates. Thus the present results and those of Deekshit et al. (2012) agree that some antimicrobial-resistant genes are “silent” in bacteria in vitro; it further provides an indication that these silent genes can spread to other bacteria or turn on in vivo, especially under selection pressure of antibiotic use (Yaqoob et al. 2007). The present study showed detection of *cat 1* gene in all the six resistant genes and in all 14 multiresistant isolates as well as most susceptible isolates. Five human isolates harbored *catA* genes while 8 of 10 that harbored *cat* gene from animal samples were susceptible to chloramphenicol in vitro. In addition, *cat 2* was detected in all the raw food isolates, *floR* was detected in all 13 florfenicol-resistant *Salmonella* isolates, and *floR* was detected in all 6 florfenicol-resistant *Salmonella* isolates. Chloramphenicol used to be the drug of choice in the treatment of *Salmonella*-related infections in Nigeria after which a survey revealed 72.4–89.2% increase from 1997 to 2007, thus limiting its therapeutic value (Akinyemi et al. 2006). Aminoglycoside-resistant isolates from clinical specimens and food of animal origin is of public health importance in developing countries because they are used to treat a wide variety of infections Davis et al. (2002). The two aminoglycoside-resistant genes *aph*(3)-IIa and *aac311a* were detected in all the three aminoglycoside-resistant isolates in the present study. Of the 14 multiresistant genes, 10 (71.4%) aminoglycoside-resistant genes aph*(3)11a* tested positive and eight tested positive for the *aac311a* gene. Chicken and clinical isolates were only positive for aminoglycoside-resistant genes and none from the clam isolates. The fact that only three isolates were resistant in vitro (one clinical and two from chicken) and susceptible aminoglycoside isolates tested positive to aminoglycoside-resistant genes is noteworthy. Thus, the present results and those of Maynard et al. (2003) indicate that some antimicrobial resistance genes are “silent” in bacteria in vitro; however, these silent genes can spread to other bacteria or turn on in vivo, especially under antimicrobial pressure. The results show antibiotic resistance in *Salmonella* spp. found in food samples from India and clinical *Salmonella* isolates in Nigeria.
